# Nutritional and metabolic management of COVID-19 intensive care patients

**DOI:** 10.1016/j.jointm.2021.01.004

**Published:** 2021-02-05

**Authors:** Pierre Singer

**Affiliations:** Department of General intensive Care, Rabin Medical Center, Beilinson Hospital, Sackler School of Medicine, Tel Aviv University, abotinsky Street, Petah Tikva 49100, Israel

**Keywords:** COVID-19, Nutrition, Intensive care, Indirect calorimetry

## Abstract

Nutritional and metabolic disturbances are observed in patients critically ill with Coronavirus disease 19 (COVID-19) patients. The aim of this review is to describe these disturbances during the progression of the disease, from the pre-intubation phase through the ventilated condition to the post extubation phase. The analysis of new data describing the prevalence of malnutrition, the modifications in energy expenditure and body composition are guiding medical nutritional therapy to prevent patients from experiencing severe energy deficit and muscle loss. Rehabilitation may be extremely prolonged and therefore, nutrition is mandatory to decrease this recondition period. This review also comments on the European Society of Parenteral and Enteral Nutrition (ESPEN) nutritional statements.

## Introduction

From the beginning of the severe acute respiratory syndrome coronavirus-2 (SARS-CoV-2) pandemic, pathophysiology of the disease is better understoodithe [1,2]. Gastrointestinal disturbances such as the occurrence of diarrhea, vomiting, abdominal distension and pain, alterations in taste and dysphagia, may all impair oral intake [3] and worsen the nutritional status. These changes have strong metabolic and nutritional consequences on the body composition and ability of the patients to overcome the disease or to efficiently recover from it. From statements mainly based on existing European Society of Parenteral and Enteral Nutrition (ESPEN) guidelines [4], more recommendations have been published based on the recent accumulated experience [5], [6], [7], [8]. This document summarizes the up-to-date knowledge of the metabolic effects and the recommended nutritional therapy of patients critically ill with Coronavirus disease 19 (COVID-19).

## Nutritional screening, assessment, and vitamin requirements

### Screening and assessment

In a recent study conducted on 523 patients with COVID-19 [9], the 211 patients admitted to the intensive care unit (ICU) were older and mainly male, and had a lower body mass index (BMI) and high-density lipoprotein (HDL) cholesterol than patients treated outside of the ICU . Forty-five percent died and they suffered more morbidities as described previously [10]. Patients with higher Nutritional Risk Score (NRS) (higher than 5 points) had a significantly increased risk of inhospital death than those with lower NRS scores (odd ratio=1.880, 95% confidence interval: 1.151–3.070), which suggest that malnutrition may be an additional risk of mortality.

Once admitted to the ICU, patients with respiratory distress not requiring invasive ventilation are generally treated with high-flow nasal cannula (HFNC) oxygen therapy or non-invasive ventilation (NIV), are suffering respiratory distress and while breathing spontaneously, are treated with oxygen supplementation up to HFNC oxygen therapy or NIV. As a result, oral intake decreases significantly and enteral/parenteral nutrition is prescribed - often times in a non-optimal fashion but not optimally [11]. This decrease in energy/protein intake is associated with a prolonged immobilization that increases catabolism and muscle loss. In an audit that recruited 268 ICU patients with COVID-19 ICU patients, Pironi et al. [12] found that 75% of the patients were at a risk of malnutrition (using NRS), and, using the GLIM classification [13], 54% of the patients were diagnosed with severe malnutrition and 35% of the patients with moderate malnutrition from among the 151 evaluated patients. Rouget et al. [14] also demonstrated the existence of a high prevalence of malnutrition (37.5%) in a general cohort of inpatients with COVID-19 according to the GLIM criteria. In an additional study, the muscle wasting was observed in all examined subjects. They suffered a fat mass loss equal to about 9% [15]. This was attributable to an unusually prolonged increase in energy expenditure(EE) which was partially attributable to fever and inflammatory status. In the initial period of the acute phase, in response to the injury, an endogenous energy production occurs, between 500 and 1400 kcal/day, which is added to the daily EE. In the late period of the acute phase, resistance to anabolism occurs, with the consumption of energy and protein reserves. Regarding muscle mass, Virgensa et al. [16] analyzed the severe inflammatory process secondary to a cytokine storm, observed mainly in elderly and obese patients with COVID-19 . Severe loss of muscle mass was observed. Immobilization is also a factor of lean mass loss.

Recently, a longitudinal observational study described EE in 22 ventilated patients with COVID-19 measured by indirect calorimetry [17]. Interestingly, measured EE increased progressively from week 1 (1568 kcal/d, range 1175–2215 kcal/d) to week 2 (1830 kcal/d, range 1465–2467 kcal/d) and week 3 (2789 kcal/d, range 1776–3262 kcal/d). In another study following 7 patients [18], an extreme hypermetabolic state (mean 4044 Kcal/day with 235.7% ± 51.7% of predicted values) with extraordinary oxygen consumption and carbon dioxide production were observed. These new observations show that the acute phase may be more prolonged and that the post-acute phase may be associated with a strong hypermetabolism.

Patients who received more oxygen therapy had a significant decrease in oral intake. Of the 63 (23.5%) patients who received medical nutrition therapy, 16 patients received oral nutritional supplements, 34 patients received enteral nutrition, and 13 patients received parenteral nutrition. The energy and protein targets were achieved according to the ESPEN guidelines for nutrition in the ICU [12].

### Vitamins and trace elements status

There is a strong association between the circulating levels of 25-hydroxyvitamin D and COVID -19 positivity rates [19] suggesting that high levels of 25-hydroxyvitamin D may protect people from contracting the virus. From the CovILD registry, [20], these findings were confirmed. “A total of 109 patients were included in the analysis eight weeks after the onset of COVID-19, a high proportion of patients presented with impaired vitamin D metabolism and elevated parathyroid hormone (PTH) levels. PTH concentrations were increased in patients who needed ICU treatment, while vitamin D levels were not significantly different between disease severity groups. Low vitamin D levels at disease onset or at eight-week follow-up were not related to persistent symptom burden, lung function impairment, ongoing inflammation, or more severe CT abnormalities.” The authors concluded that vitamin D deficiency is frequent among COVID-19 patients but not associated with disease outcomes.

Selenium deficiency is associated with increased COVID-19 mortality [21]. Patients living in areas with poor baseline supply, and with preexisting comorbidities such as lung diseases have an elevated risk of severe selenium deficiency and are increasingly exposed to COVID-19 [21].

## Nutritional management in ICU patients infected with SARS-CoV-2

**Fig. 1:**
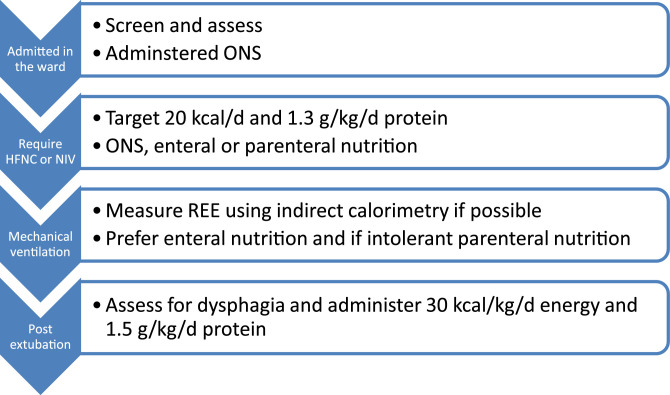
Flow chart of medical nutritional support according to the progression of the disease. ONS: Oral nutritional therapy; HFNC: High flow nasal cannula oxygen therapy; NIV: Non invasive ventilation; REE: Resting energy expenditure.

From the time the recent ESPEN guidelines on nutritional therapy were released [4], numerous studies have described the nutritional therapy according to the respiratory support allocated to the ICU patient [5], [6], [7], [8] (see Fig. 1).

### Preintubation period

In the first wave, patients with COVID-19 with early signs of respiratory distress were promptly treated with mechanical ventilation, based on the fear of disseminatoin of the virus if non-invasive therapy was used. During the second wave, oxygen flow nasal cannula (FNC), HFNC or NIV using a mask or a helmet were prescribed for a longer period of time to avoid mechanical ventilation.

The *Statement Consideration number 7* [4] suggested that in nonintubated ICU patients with COVID-19 who do not reach the energy target with an oral diet, oral nutritional supplements (ONS) should be considered first and then enteral nutrition treatment should be given. The introduction of a nasogastric or naso-duodenal tube should be performed with caution because many bleeding episodes have been reported, mainly if the patient were to receive full anticoagulation. Blind placement of the duodenal tube has been proposed to decrease the need for gastroscopy or fluoroscopy [22].

If there are limitations for the enteral route, it could be advised to prescribe peripheral/central parenteral nutrition in the population that does not reach energy-protein target by oral or enteral nutrition. This approach has been confirmed by the French and Chinese positions papers [5,23]. The use of NIV may induce stomach dilatation and inadequate implementation of enteral feeding, which results in partial undernutrition. In these cases, peripheral or central parenteral nutrition should be considered. The use of FNC and HFNC may decrease oral alimentation. Few studies described the implementation of nutritional support when this technique is used. We recently described a significant increase in the number of minor and massive gastroesophageal refluxes using HFNC that impairs calorie and protein intake [24]. The assessment of nutrient intake should be initiated.

### Ventilated period

The exact timing to initiate mechanical ventilation is still unknown [25]. The ESPEN COVID-19 statements recommend [4]:

*Recommendation Statement Consideration 8*: Enteral nutrition(EN) should be started through a nasogastric tube; post-pyloric feeding should be performed in patients with gastric intolerance after prokinetic treatment or in patients at high-risk for aspiration; and the prone position per se does not represent a limitation or contraindication for EN. Patient EE should be determined to evaluate energy needs by using indirect calorimetry when available. The use of indirect calorimetry was discussed by scientific societies because of the fear of virus spread [6]. The practical guidance for the safe use of indirect calorimetry has been recently proposed [26], which allows a larger use of indirect calorimetry in this specific population. As described above, Whittle et al. [17] performed a longitudinal study of measured EE in 22 ventilated patients with COVID-19. Despite the cytokine storm, the first week was characterized by lower EE (15–20 kcal/kg actual body weight in BMI < 30 and adjusted body weight in obese subjects after 1 week), hypermetabolism increased further and during the third week, it reached 150% of the predicted resting EE. Predictive equations such as the Harris Benedict equation were found to be inaccurate. A large variability in the measurements was also observed. If calorimetry is not available, carbon dioxide production derived from the ventilator will give a better evaluation of EE than predictive equations [4].

Energy administration should be guided by the recent ESPEN ICU Nutrition guidelines [27] and suggest that “hypocaloric nutrition (not exceeding 70% of EE) should be administered in the early phase of acute illness with increments of up to 80–100% after day 3. If predictive equations are used to estimate the energy need, hypocaloric nutrition (below 70% estimated needs) should be preferred over isocaloric nutrition for the first week of ICU stay due to reports of overestimation of energy needs”. Interestingly, in a study, including patients with COVID-19 [9], those who died were started later on nutritional support and received more parenteral nutrition (62.1% *vs*.10.3%, *P*<0.001).

Protein requirements: The ESPEN statements recommend to deliver 1.3 g/kg protein equivalents per day progressively. This statement is endorsed by Thibault et al. [5]. The standing American Society of Parenteral and Enteral Nutrition (ASPEN) recommendations for protein as 1.2–2.0 g/kg of bodyweight is also relevant in this case [28]. Patients with COVID-19 are often bedridden for a prolonged period and suffer from a hyperinflammation state. Both conditions lead to a loss of skeletal muscle mass and a hyperinflammatory condition. To enhance skeletal muscle anabolism, physical activity and mobilization are recommended.

*Recommendation Statement Consideration 9*: In ICU patients who do not tolerate full dose EN during the first week in the ICU, initiating parenteral nutrition (PN) should be weighed on a case-by-case basis. PN should not be started until all strategies to maximize EN tolerance have been attempted.

## Limitations and precautions

Patients with uncontrolled life-threatening hypoxemia, hypercapnia or acidosis, and uncontrolled shock should not be fed enterally or parenterally until the shock is controlled with fluids and vasopressors or inotropes [27]. Progression to full nutrition should be performed cautiously in these patients. Refeeding syndrome may occur, mainly if patients have been without adequate nutritional intake for a long period of time, in fear of aspiration or respiratory decompensation that requires intubation. Finally, blood glucose should be maintained at target levels between 6 and 8 mmol/l, along with monitoring of blood triglycerides and plasma levels of potassium, phosphate, and magnesium.

Prone position has become a frequent and valid technique to improve oxygenation [29] and nutritional therapy has been reviewed recently [30]. Most of the studies show that early administration of enteral nutrition, which progresses to target is safe and well tolerated even at rates up to 85 mL/h at day 4. Prone position includes maintaining the head elevation (reverse Trendelenburg) at a 10–25° [31], and evaluating and managing risk factors on an individual basis. If enteral nutrition is not tolerated, parenteral nutrition should be considered. Large gastric residue is not more frequent in prone or supine position [31].

### Post-mechanical ventilation period and dysphagia

After prolonged intubation, swallowing problems and dysphagia occur and limit oral nutrient intake. Two hundred and eight patients were assessed for dysphagia, (102 post ICU patients of which 82 were tracheostomized) [32]. The time from extubation to oral intake was 5.3 ± 2.3 days and from tracheostomy to oral intake 14.8 ± 6.6 days. The majority of patients regained near normal swallow function prior to discharge, regardless of intubation duration or tracheostomy status after intensive speech therapy. Therefore, a whole systems approach is recommended to manage dysphagia associated with COVID-19, including medical, surgical, nursing, therapy, and education teams is therefore recommended.

*Recommendation Consideration Statement 10:* In ICU patients with dysphagia, texture-adapted food can be considered after extubation. If swallowing is proven unsafe, EN should be administered. In cases with a very high aspiration risk, postpyloric EN or, if not possible, temporary PN during swallowing training with removed nasoenteral tube can be performed.

### Post COVID-19 chronic illness

According to Curci et al. [33], 32 post-acute patients with COVID-19 (22 male and 10 female), with mean age of 72.6 ± 10.9 years had a Barthel index of 45.2 ± 27.6. All were dyspneic and only 14  (43.7%) patients were able to walk. The 6-minutes walk trial was feasible in 6 (18.8%) patients with a mean distance of 45.0 ± 100.6 m. In a single center study [34] of 143 patients who recovered from COVID-19, 44% reported decreased quality of life and 87% of the patients reported persistent symptoms, including dyspnea, chest pain, cough, fatigue, and joint pain. The post COVID-19 chronic illness has many common features with the Persistent Inflammatory and Catabolic State (PICS) [35]. Loss of skeletal muscle mass and muscle function may be tremendous and a major problem, mainly in older adults and comorbid patients who are more prone to present with pre-existing catabolic conditions and impaired skeletal muscle mass and function. Bangash et al. [36] suggested that the use of β-adrenergic blockers as anti-arrhythmics with proven benefit in the cardiovascular arena, also has the potential ability to reduce the systemic metabolic rate and catabolism, and modulate immune dysfunction [37]. Finally, the authors propose nutritional supplements, such as niacin and folic acid to intervene and improve the muscle and the cardiovascular system, mediated through effects on DNA methylation and cellular energetics [38], [39].

## Conclusions

At each step of the progression of the disease, ICU patients with COVID-19 should be carefully evaluated in terms of malnutrition risk and medical nutritional support. The tremendous challenges posed by the continuous clinical changes requires adequate answers at each stage. A delay will induce more energy and protein deficit, longer length of stay, and rehabilitation for the survivors. Metabolic control and nutritional support are a cornerstone in the management of critically ill patients with COVID-19.

## Conflicts of Interest

The author declares that he has no known competing financial interests or personal relationships that could have appeared to influence the work reported in this paper.
